# A Plea for Studying Qualitative Individual Differences by Default

**DOI:** 10.5334/joc.172

**Published:** 2021-08-27

**Authors:** Isabel Thielmann

**Affiliations:** 1 Department of Psychology, University of Koblenz-Landau, Fortstraße 7, 76829 Landau, Germany

**Keywords:** Decision making, Judgment, Motivation

## Abstract

I see great potential in the approach proposed by Rouder and Haaf. First, using an example from unethical decision making, I demonstrate that considering quantitative individual differences alone can make us overlook important psychological phenomena that are only visible at the individual level. Thus, the study of quantitative individual differences should, by default, be complemented by investigation of qualitative individual differences. Second, having powerful tools to study qualitative individual differences in cognition has great potential to advance personality science. Recently, personality psychologists are increasingly working towards obtaining a better understanding of the processes that underlie the expression of personality in behavior. The toolbox provided by Rouder and Haaf may add to this research in meaningful ways.

Rouder and Haaf pose the question whether it is useful to consider qualitative individual differences in cognition in addition to commonly studied quantitative individual differences. My answer to this question is clearly “yes”. First, investigating qualitative individual differences may not only provide vital information on the generality of certain phenomena; considering quantitative individual differences (i.e., averages) alone can make us overlook important phenomena that are only visible at the individual level – with potentially far-reaching implications for theory, research, and practice. Second, having powerful tools to study qualitative individual differences has great potential for personality science because it can help us understand the processes that underlie the expression of stable individual differences in behavior – an issue that has received (far too) little attention in prior work. In this commentary, I will substantiate both these propositions.

## Focusing on Quantitative Individual Differences Alone Can Lead to False Conclusions

Rouder and Haaf demonstrate that qualitative individual differences may exist in addition to quantitative individual differences in certain phenomena. For example, they illustrate that although most individuals show a rightward bias in an orientation task, some individuals show a leftward bias. Although such effects alone may warrant the consideration of qualitative individual differences by default, a more compelling case is made by examples where the mere focus on quantitative individual differences would obscure the influence of a variable on behavior.

Such an example is provided by the influence of incentives on unethical decision making. According to an expected utility account, individuals should trade off the potential benefits of unethical behavior (e.g., dishonesty) against the potential costs and their respective probability of occurrence (Becker, 1968). Thus, all else being equal, unethical behavior should increase with increasing benefits. Contrary to such an economic approach, however, meta-analyses consistently show that benefits have no reliable effect on dishonest behavior when considered on the aggregate level (Abeler et al., 2019; Gerlach et al., 2019). That is, even in a situation where people do not have to fear any sanctions because lying is completely anonymous, only a fraction of people (approx. 25–30%) lie, and this fraction is essentially the same irrespective of whether a lie is worth $1, $5, or $20. One may therefore – prematurely – reject the expected utility account altogether and conclude that incentives are irrelevant for unethical decision making.

Crucially, inspection at the individual (i.e., within-subjects) level tells a different story. Using a multi-trial cheating paradigm in which participants were repeatedly confronted with the decision to lie for different benefits that varied between 5€ and 105€, we found that incentives do have an effect, but this effect strongly differs between individuals (Hilbig & Thielmann, 2017). Whereas some individuals became *more* willing to lie with increasing benefits (i.e., “corruptibles”), others became *less* willing to lie with increasing benefits (i.e., “small sinners”), and even others were similarly (un)willing to lie for smaller and larger benefits (i.e., “honest individuals” and “brazen liars”, respectively). On the aggregate level, in turn, we once again found no evidence for an effect of incentives on dishonesty whatsoever, thus confirming previous meta-analytic findings.

As this example demonstrates, conclusions derived from the sole consideration of quantitative individual differences may be premature and, in fact, wrong. Thus, even in the absence of quantitative individual differences, it may be worthwhile to investigate qualitative individual differences, simply because different effects at the individual level may cancel each other out and obliterate effects when regarded at the aggregate level. In turn, identifying different patterns of cognition and behavior at the individual level can have important theoretical implications. In the case of unethical decision making, our findings suggest that an expected utility account can indeed describe the behavior of some (corruptible) individuals, but it falls short of describing the behavior of others (e.g., small sinners, honest individuals) unless additional presumptions are made (e.g., adding psychological costs of lying to the equation, beyond material costs; Thielmann & Hilbig, 2019). In general, the findings emphasize that theories of unethical decision making should be able to account for the diversity in individuals’ reactions to changes in material benefits.

## Studying Qualitative Individual Differences Can Add to Personality Science

Another reason why we should by default consider qualitative individual differences in cognition is that the approach can significantly advance personality science. For decades, a – or arguably *the* – dominant question in personality psychology has been how stable individual differences can best be described in terms of broad trait taxonomies (Ashton & Lee, 2020) and to what extent these broad traits, as well as their constitutive lower-level aspects, can predict certain behavioral outcomes (Ozer & Benet-Martínez, 2006; Zettler et al., 2020). This focus is currently changing. Recently, many have called for moving personality science forward to questions relating to the cognitive, affective, and motivational *processes* that underlie the expression of personality in behavior (Back & Vazire, 2015; Baumert et al., 2017; Quirin et al., 2020). Such research may ultimately help us understand whether individual differences in the processing of information can *explain* why certain traits relate to certain behaviors. The toolbox provided by Rouder and Haaf can significantly add to this process-oriented work by uncovering fine-grained individual differences in various phenomena related to human cognition.

## Conclusion

Taken together, I concur with Rouder and Haaf that “group- or population-level averages are fine as a start, but are inherently limited”. We know that individuals differ in various meaningful ways. Thus, if we want to truly understand human behavior, we need to abandon a mere focus on the aggregate level but start to establish the investigation of individual differences as standard. Rouder and Haaf make a significant step in this direction. Their approach and toolbox have the potential to contribute to different fields and research streams within psychology that emphasize the significance of the individual.
